# Distinct Difference in Sensitivity of NIR vs. IR Bands of Melamine to Inter-Molecular Interactions with Impact on Analytical Spectroscopy Explained by Anharmonic Quantum Mechanical Study

**DOI:** 10.3390/molecules24071402

**Published:** 2019-04-10

**Authors:** Justyna Grabska, Krzysztof B. Beć, Christian G. Kirchler, Yukihiro Ozaki, Christian W. Huck

**Affiliations:** 1 Institute of Analytical Chemistry and Radiochemistry, Leopold-Franzens University, Innrain 80/82, CCB-Center for Chemistry and Biomedicine, 6020-Innsbruck, Austria; justyna.grabska7@gmail.com (J.G.); Christian.Kirchler@uibk.ac.at (C.G.K.); Christian.W.Huck@uibk.ac.at (C.W.H.); 2 Faculty of Chemistry, University of Wrocław, F. Joliot-Curie 14, 50-383 Wrocław, Poland; 3 Department of Chemistry, School of Science and Technology, Kwansei Gakuin University, Sanda, Hyogo 669-1337, Japan; ozaki@kwansei.ac.jp

**Keywords:** melamine, FT-IR, NIR spectroscopy, quantum chemical calculation, anharmonic calculation, overtones, combination bands

## Abstract

Melamine (IUPAC: 1,3,5-Triazine-2,4,6-triamine) attracts high attention in analytical vibrational spectroscopy due to its misuse as a food adulterant. Vibrational spectroscopy [infrared (IR) and Raman and near-infrared (NIR) spectroscopy] is a major quality control tool in the detection and quantification of melamine content. The physical background for the measured spectra is not interpreted in analytical spectroscopy using chemometrics. In contrast, quantum mechanical calculations are capable of providing deep and independent insights therein. So far, the NIR region of crystalline melamine has not been studied by quantum mechanical calculations, while the investigations of its IR spectra have remained limited. In the present work, we employed fully anharmonic calculation of the NIR spectrum of melamine based on finite models, and also performed IR spectral simulation by using an infinite crystal model—periodic in three dimensions. This yielded detailed and unambiguous NIR band assignments and revised the previously known IR band assignments. We found that the out-of-plane fundamental transitions, which are essential in the IR region, are markedly more sensitive to out-of-plane inter-molecular interactions of melamine than NIR transitions. Proper description of the chemical surrounding of the molecule of melamine is more important than the anharmonicity of its vibrations. In contrast, the NIR bands mostly arise from in-plane vibrations, and remain surprisingly insensitive to the chemical environment. These findings explain previous observations that were reported in IR and NIR analytical studies of melamine.

## 1. Introduction

Melamine (IUPAC: 1,3,5-Triazine-2,4,6-triamine) has wide industrial importance, nowadays being used e.g., in the manufacture of polymers and resin [1], concrete [2], flame-resistant materials [3], and it may be utilized in the production of nanomaterials (e.g., *N*-doped carbon nanotubes) [4]. In the past, it was even more widely applied in industry and agriculture [5]. It was unfortunate that melamine has become infamous worldwide as a dairy adulterant after it caused a milk safety crisis in 2008 with severe casualties (290,000 people affected with 51,900 hospitalized in China only) [6]. That event had a global impact on the food industry, food production, supply chains, and corresponding legal regulations [7,8]. It strongly echoed in the field of food quality control, leading to a strong stimulus for development and the adaptation of adequate analytical routines [8,9,10]. A number of other food safety incidents in recent years have induced particular pressure on this area of analytical chemistry [11,12]. The methods that are based on vibrational spectroscopy [infrared (IR), Raman and near-infrared (NIR)] have become particularly important elements of this effort in controlling the food safety at every stage of its production and supply [13,14,15].

Vibrational spectroscopy stands out as a non-invasive, widely applicable, low-cost, and quick time-to-result analytical method. Therefore, it combines advantages that are highly valued in analytical chemistry. Despite being grouped together, the key differences among these three kinds of techniques should be noted. IR (4000–400 cm^−1^) spectroscopy elucidates chemical information from the fundamental vibrational transitions. In contrast, the signal that was measured in NIR (10,000–4000 cm^−1^) spectroscopy originates from the excitations of higher quanta transitions, mostly first overtones and binary combinations [16,17,18]. Raman spectroscopy also provides information regarding fundamental vibrations, but through a distinctly different working principle than IR spectroscopy. The differences in the wavelength regions and underlying physical background translate into distinct differences in the instrumentation and applicability of these methods. Each of these techniques offer unique advantages, but for the detection/quantification of melamine content in milk, NIR spectroscopy may be favored [9]. A number of factors contribute to this fact [19]. Higher sample volume resulting from low NIR absorptivity of matter in general, and water, in particular, allows for more straightforward measurement of transmittance or reflectance of the milk sample. The same may be achieved with ATR-IR (Attenuated Total Reflection IR) approach; however, the fiber probe compatibility of NIR instrumentation gives it superior flexibility in high-volume analysis. NIR spectroscopy also benefits from the largest tolerance for the sample inhomogeneity. Raman spectroscopy is suitable for the measurement of aqueous samples, but for similar analytical applications Raman instrumentation is often more expensive. Finally, in NIR spectroscopy, a strongly stimulated development for miniaturization [20,21] has culminated in highly affordable micro-spectrometers, which are available under 300 USD nowadays [22]. In a strict application to melamine detection/quantitation, all three techniques have been successfully used in the literature 9]. However, NIR spectroscopy demonstrates the best analytical performance in this case [9], on top of its practical advantages [23,24].

The nature of NIR spectra (overlaying overtones and combinations) [16,17,18] largely limits their interpretability [25,26]. Chemometric methods do not provide physical insights on the analyzed sample, and NIR spectroscopy is often used as a “black-box”. In contrast, for IR and Raman spectroscopy, this limitation is less severe due to its more simple spectra with milder fundamental band overlapping [27,28]. Coincidently, quantum chemistry offers affordable methods (harmonic approximation) for the adequately accurate simulation of IR and Raman spectra [29,30]. In contrast, prediction of NIR bands require resource intensive anharmonic methods [31,32]. The difference in resource demand (harmonic vs. anharmonic approximation) is significant, and theoretical NIR studies of complex molecules have only recently appeared [33,34,35,36,37,38]. Lately, we have reported the quantum mechanical calculations of NIR spectra of various molecules in solution, liquid, and solid state, including short- [34], medium- [35], and long-chain [36] fatty acids. These studies could have been used, e.g., for the interpretation of the meaningful NIR bands that influence the chemometric models used in quantification of the content of phytopharmaceutical compounds in natural drugs [37,38,39]. On the other hand, simulations of IR spectra in crystalline phase that use a proper representation of infinite crystal lattice by a three-dimensional (3D) periodic model remain equally rare.

In the literature so far, quantum chemical calculation vibrational studies of melamine have been limited to finite models and harmonic approximation [40,41]. Mircescu et al. have harmonically calculated IR and Raman spectra of melamine [40]. They have used the single molecule model, and cluster of 10 melamine molecules. They have compared the spectra that were calculated with these two approaches and judged that the 10 molecule cluster model leads to a better quality of the calculated IR and Raman spectra of melamine. They concluded that the hydrogen-bonding of melamine in crystal needs to be taken into account in order to yield accurate calculated vibrational spectra. Accordingly, the calculations that are based on the 10 molecule cluster have led to much improved simulated IR and Raman spectra. Yuan et al. have drawn similar conclusions [41] in their quantum mechanical calculations of IR spectra of melamine. They have used a single molecule model, a four-molecule cluster with two hydrogen-bonds, and a large cluster consisting of 32 molecules of melamine featuring 30 intermolecular hydrogen-bonds. They have compared the IR spectra that were calculated on the basis of these models and concluded that proper representation of the hydrogen-bonded structure of melamine is essential in improving the quality of the calculated IR spectrum [40,41].

Therefore, the earlier studies [40,41] have recognized the importance for spectra calculation of the proper description of the chemical neighborhood in crystalline melamine, in particular, the hydrogen bonding network. However, the methodology in these studies has been limited to finite models, clusters of melamine molecules (10 to 32 molecules). Although improved vs. single molecule models, the finite boundary of these model clusters has led to the distortions of the molecular structure as compared with the structure of the crystal lattice of melamine. This has resulted in a number of “phantom bands” appearing in the calculated IR and Raman spectra, which could not be observed in the experimental spectra [40,41]. Additionally, these previous studies have been limited to harmonic approximation, which made any calculations of NIR bands unavailable. Accordingly, there are no NIR spectra simulations of melamine of any kind reported so far. On the other hand, melamine was intensively focused on in analytical near-infrared reflectance spectroscopy (NIRS) [9,42,43,44,45,46]; however, these studies have not been able to derive insightful physicochemical information on melamine.

The purpose and novelty of the present study is to explore NIR vs. IR spectra correspondences in crystalline melamine. To achieve this, for the first time, we employ anharmonic quantum mechanical calculations of NIR spectra of melamine, by using two different approaches, which we directly compare. Moreover, we improve the previous investigations of IR spectra in a well-defined crystalline lattice by employing an infinite three-dimensional (3D) periodic model of the crystalline melamine, for the first time as well. This yields more accurate calculated IR spectrum, but it also is essential in obtaining good comprehension of a number of relevant effects. In example, the impact of anharmonicity may become well separated from the influence of the chemical neighborhood. The distinct difference in the importance of inter-molecular interactions for the accurate reproduction of IR and NIR transitions of melamine is found and explained. This means that, in contrast to IR bands, the accurate reproduction of NIR bands requires significantly less attention in describing the long-range, and in particular, inter-plane, interactions in crystalline melamine.

## 2. Results and Discussion

### 2.1. Experimental and Simulated IR Spectra of Crystalline Melamine

**Figure 1:**
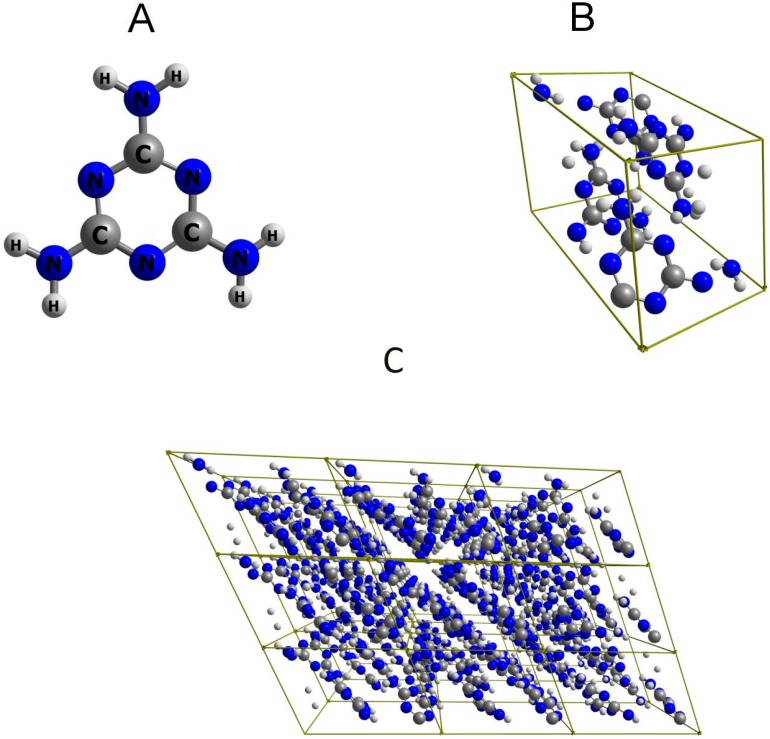
Molecular structure of melamine. (**A**) single molecule; (**B**) the content of a unit cell; (**C**) 3 × 3 × 3 supercell. The structure after optimization (B3LYP\Gatti) is presented in Figure S1 (Supplementary Materials).

**Figure 2:**
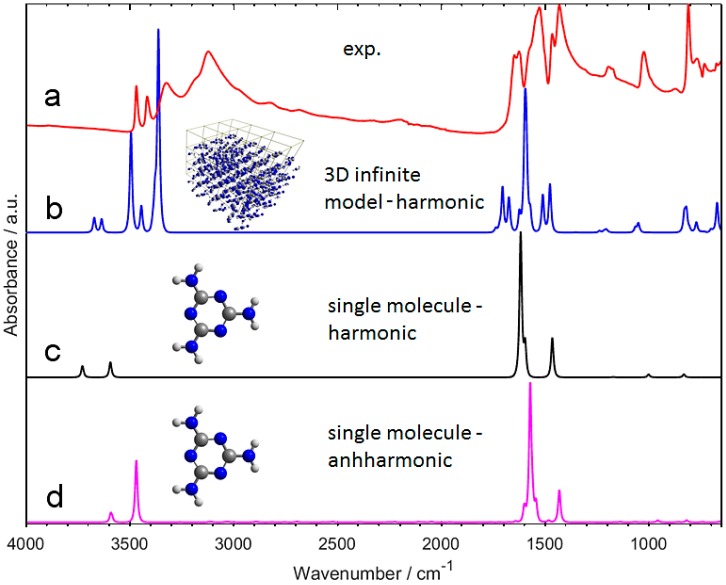
Comparison of the experimental Attenuated Total Reflection IR (ATR-IR) spectrum of polycrystalline melamine (**a**) with the calculated spectra (**b**–**d**). The spectra were calculated in harmonic ((**b**) B3LYP/Gatti for periodic (3D, infinite) model; (**c**) B3LYP-GD3BJ/SNST for single molecule model) and anharmonic ((**d**) GVPT2//DFT-B3LYP-GD3BJ/SNST) approximation. For the calculated spectra, no scaling was applied in this figure.

The simulation of the IR spectrum of melamine in polycrystalline state requires a proper representation of the long-range ordered structure (Figure 1). There exists a decisive decrease in the accuracy for the IR spectrum calculated on the basis of finite model, even in anharmonic approximation (Figure 2). Such spectra are markedly poor and numerous bands are missing (Figure 2C,D). In contrast, the spectrum that was calculated for infinite (3D periodic) model (Figure 2B) correctly reproduces all of the major experimental bands (Figure 2A). The overestimation of the calculated peak positions, particularly noticeable above 3000 cm^−1^, likely results foremost from neglecting the anharmonic effects that are typically strong in the X-H stretching region. However, this may be accurately corrected by employing wavenumber scaling. This observation may appear obvious; however, in light of NIR simulations (as discussed in Section 2.2 and Section 2.3), it leads to farer-reaching conclusions. In Section 2.4, we will explore this topic in detail.

**Figure 3:**
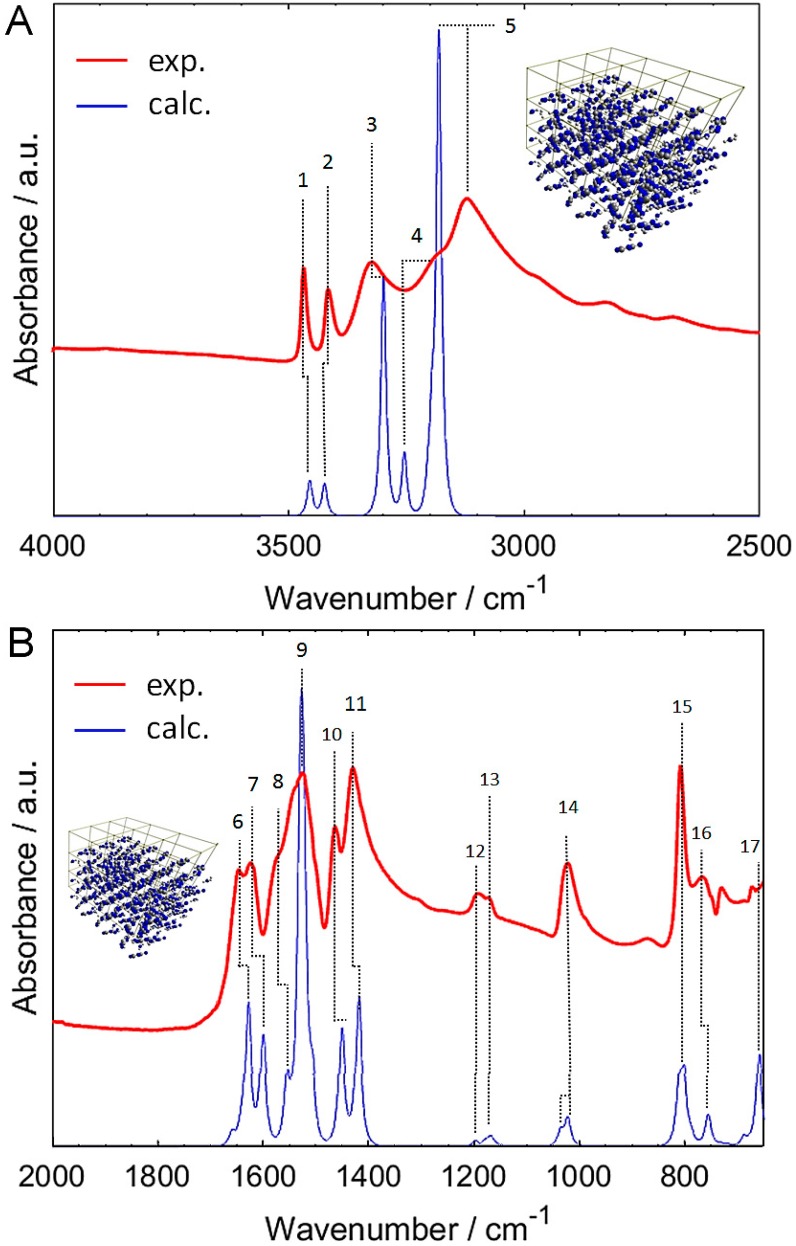
Band assignments in the ATR-IR spectrum of polycrystalline melamine based on scaled periodic//B3LYP/Gatti calculated spectrum. Band numbers correspond to those that are presented in Table 1. (**A**) 4000–2500 cm^−1^ region; (**B**) 2000–650 cm^−1^ region.

Therefore, the discussion of the IR bands will be based on harmonic periodic system calculations. The neglecting of anharmonicity in the case of periodic system calculations did not decrease noticeably the agreement with the experimental spectrum. We have carried out two separate calculations of IR spectra for the lattice model (B3LYP/Gatti and B3LYP/TZVP; Figures S2 and S3 in Supplementary Materials). The differences between these two simulated spectra are qualitatively negligible, with the exception of the low-lying bands in the region of 900–650 cm^−1^ (Figure S3 in Supplementary Materials). Accordingly, the overhead computing cost that is introduced by the larger TZVP basis set did not return any profit in the case of melamine. The accuracy of scaled B3LYP/Gatti allows for unambiguous band assignments in the entire 4000–650 cm^−1^ region of crystalline melamine (Figure 3A,B; Table 1).

A good comprehension of all IR bands in the crystalline melamine was accomplished; the resulting assignments are presented in Figure 3A,B and in Table 1. The upper IR region (X-H stretching region) is mostly populated by *ν*_as_NH_2_ bands; three of them are separated, while the fourth one (at ca. 3188 cm^−1^) overlaps with the neighboring strong *ν*_s_NH_2_ peak. That single *ν*_s_NH_2_ band at 3122 cm^−1^ has the highest intensity in this region. These features are very well reflected in the calculated spectrum. The broadening that was observable just below 3000 cm^−1^ in the experimental spectrum Figure 3A) originates from the strong anharmonic effects that occur because of the long-range ordering of hydrogen-bonded melamine molecules, which exists in the crystal lattice [47,48]. These effects have well-known impact on IR spectra [47,48,49,50].

The lower IR region (fingerprint region; 1700–650 cm^−1^ in the present case; Figure 3B) of melamine features rather well separated bands. The most notable group of the intense bands in the region of 1650–1430 cm^−1^ primarily arises from in-plane NH_2_ deformations (scissoring and rocking modes of NH_2_; in-plane ring modes and C-N(H_2_) stretching modes). The internal coordinates corresponding to these vibrations are rather highly mixed (Table 1). In contrast, the bands appearing at 1194 and 1174 cm^−1^ stems from relatively “clean” NH_2_ rocking modes. However, the intensities of those two bands are weak. These observations will find good confirmation in the subsequent analysis of the NIR spectrum of melamine. The next band (1024 cm^−1^) has a moderate intensity and it corresponds to two transitions; *δ*_rock_NH_2_ mixed with *δ*_ip_ring (at calc. position of 1035 cm^−1^) and *δ*_rock_NH_2_ mixed with *ν*C-N(H_2_) and *δ*_ip_ring (at calc. position of 1021 cm^−1^). The very strong band at 810 cm^−1^ originates from the out-of-plane deformations. The transitions corresponding to less mixed *δ*_rock_NH_2_ and *δ*_rock_NH_2_ give rise to weak bands at 768 and 675 cm^−1^ (calc. 755 and 661 cm^−1^), respectively (Figure 3B and Table 1). Thus, the mixing of internal coordinates can consistently be noted for the corresponding bands with stronger intensities.

### 2.2. Experimental and Simulated NIR Spectra of Crystalline Melamine

**Figure 4:**
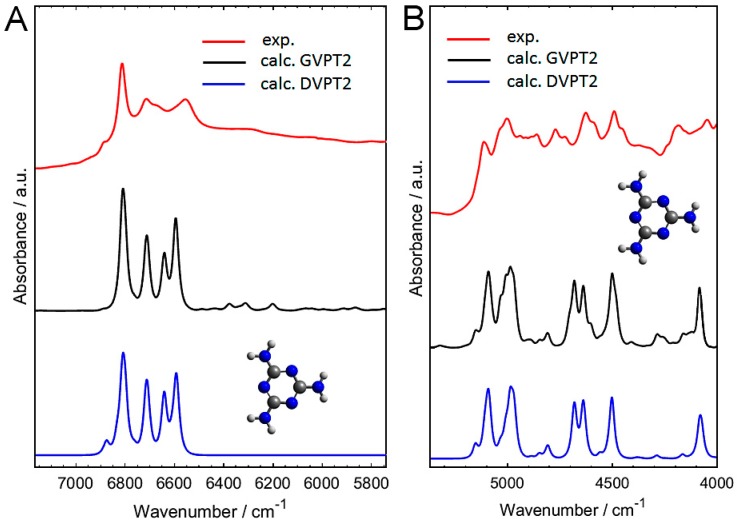
Comparison of the experimental diffuse reflectance near-infrared (NIR) spectrum of polycrystalline melamine with the calculated spectra. The spectra were calculated at B3LYP-GD3BJ/SNST level for an isolated molecule in two different anharmonic approximations (DVPT2 and GVPT2). In the GVPT2 calculation second overtones and ternary combinations were also included. (**A**) 7150–5750 cm^−1^ region; (**B**) 5400–4000 cm^−1^ region.

In decisive contrast to the IR region (Section 2.1), and as evidenced in Figure 4, the NIR bands of crystalline melamine are accurately reproduced on the basis of a finite model. In this case, even the calculations that are based on a single molecule of melamine provide good agreement between the calculated and experimental spectra (Figure 4). If further studies will allow for generalizing this observation, a lower requirement for the model complexity in modeling of NIR spectra could open other opportunities for refining the theoretical approach. For example, a hybrid approach combining higher-level harmonic computations augmenting DVPT2/GVPT2 anharmonic analysis could be used. Barone and co-workers have reported evidences of accurate and affordable hybrid B3LYP(harmonic)/B2PLYP(anharmonic) computations [51,52], as also seen in our previous studies [53].

**Figure 5:**
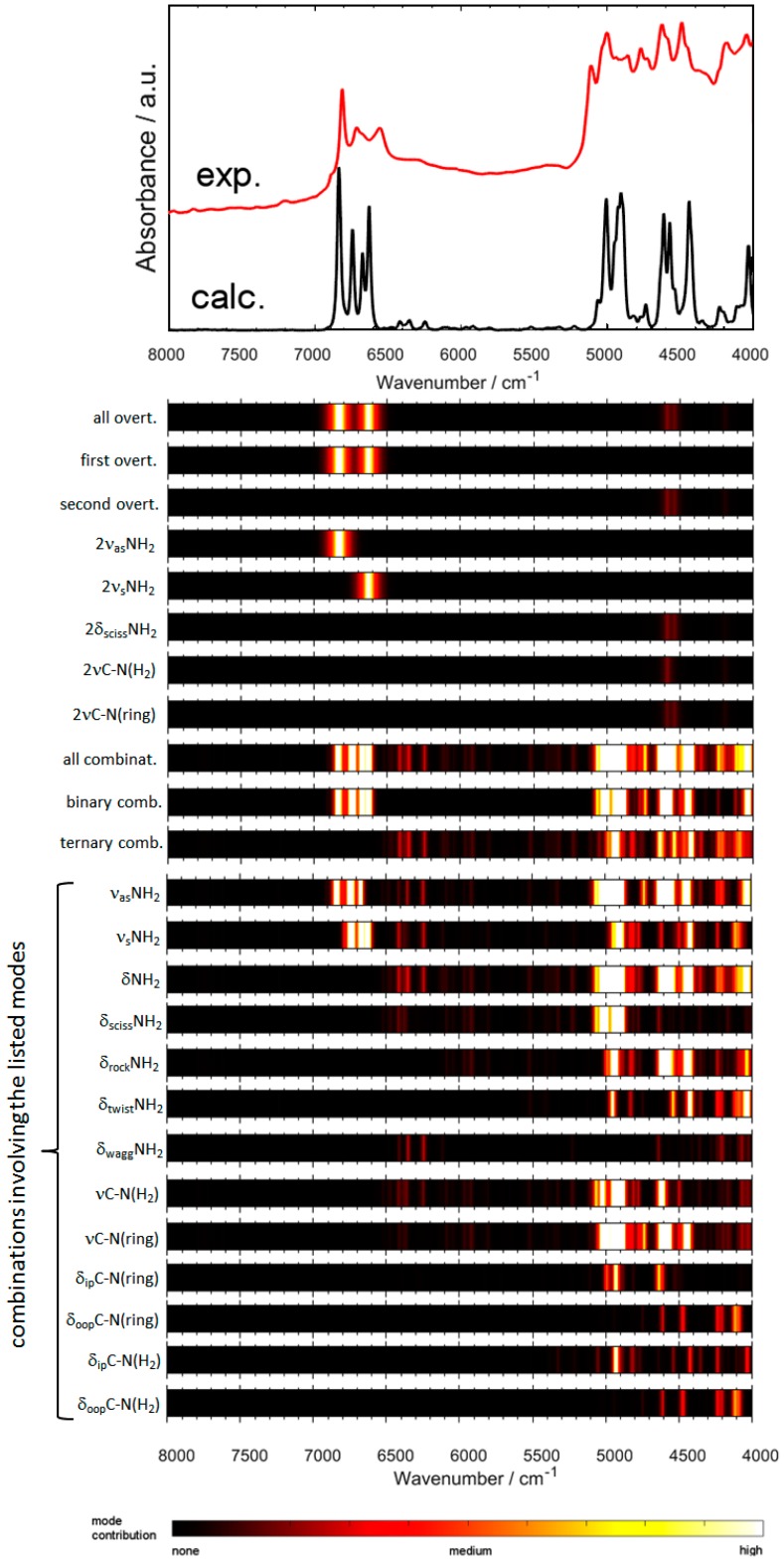
Analysis of the contributions to NIR spectrum of polycrystalline melamine based on the calculated spectrum (GVPT2//B3LYP-GD3BJ/SNST).

The principle difference between the DVPT2 and GVPT2 approaches is the treatment of vibrational resonances; the latter method features an advanced correction for Fermi and Darling-Dennison resonances by the variational method [54]. The improvement does not seem to significantly affect the major NIR bands of melamine (Figure 4). However, improved method allows for calculations of three quanta transitions (i.e., second overtones, 3*ν*; and ternary combinations, 2*ν*_x_ + *ν*_y_, and *ν*_x_ + *ν*_y_ + *ν*_z_). The addition of these minor bands slightly enhances the agreement, for example, below 6500 cm^−1^ and in the region of 4400–4150 cm^−1^ (Figure 4 and Figure 5). This observation remains similar to our previous estimations that were based on the anharmonic study of methanol molecule and its deuterated isotopomers [55]. We have therein concluded that the bands due to three quanta transitions are only responsible for minority (ca. 20%) of the NIR spectra of methanol [55]. These higher order bands are weak, overlapping bands, and the corresponding spectral information is “diffused” along the wavenumber axis [55]. A similar case may be reported for melamine, and it is probably shared by other molecules. There are some exceptions, e.g., in the literature, 3*ν*C=O has been reported to appear near 5150–5160 cm^−1^ in gas phase (and near 5122–5076 cm^−1^ in various solvents), as a well resolved band in some molecules with a C=O group [56,57]. No such exceptions were observed for melamine, and close inspection of the theoretical spectra confirms that the majority of the experimental bands were reproduced by both approximations (Figure 4). The more comprehensive, but also resource intensive, calculations of additional three quanta transitions of melamine allowed for explaining finer features that were observed in its NIR spectrum (Figure 4). Primarily, the influence of higher order bands may be seen throughout the 6500–5200 cm^−1^ region and in the vicinity of 4300–4100 cm^−1^ (Figure 4A,B). In the former case (6500–5200 cm^−1^ region), these bands remain very weak. For the latter (4300–4100 cm^−1^ region), there appears a marked band overlapping that gives rise to a broadened feature of moderate intensity at 4300–4200 cm^−1^, and also a similar one appearing at the high-frequency wing of the ~4090 cm^−1^ band (Figure 4B). The present study of NIR spectra of crystalline melamine may be compared with our earlier calculations of NIR spectra of medium-chain fatty acids [35]. In contrast to the previously observed significance of the hydrogen-bonding interaction for the NIR region of crystalline sorbic acid [35], NIR bands of melamine reveal surprisingly low sensitivity to the hydrogen-bonding. This occurs despite the fact that melamine interacts strongly and it forms multiple hydrogen-bonds in crystalline states, as recently demonstrated by Yuan et al. [41]. We will discuss in detail the reasons for such distinctiveness of NIR region of melamine in Section 2.3.

The agreement with the experimental spectrum is slightly lower in the 5200–4000 cm^−1^ region, as the level of band overlapping is very high there. In contrast, the upper NIR region (ca. 6900–6450 cm^−1^) was accurately reproduced in the calculation (Figure 4A). The first overtones and the binary combinations bands of NH_2_ stretching modes populate this region (as the primary contributions). Similar to other kinds of X-H vibrations (e.g., OH), these modes are expected in the literature to be sensitive to the molecules’ chemical neighborhood, e.g., prone to red-shifting in hydrogen-bonded complexes. This effect has been clearly observed for alcohols [58] or in our recent NIR investigation of thymol [37]. Surprisingly, in the case of melamine it may be concluded that the first overtones and binary combinations bands of NH_2_ stretching modes are properly reproduced, even in the simplified case of an isolated molecule, for which the inter-molecular interactions are not reflected (Figure 4A).

### 2.3. An In-Depth Analysis of the Origin of NIR Bands of Crystalline Melamine

Numerous overlapping bands populate NIR spectra of even relatively simple molecules [16,31]. This makes their detailed analysis difficult, and the interpretation of the leading contributions is not as straightforward as it is for the corresponding IR spectra. To better elucidate these influences and to present them in a clear manner, we have developed a density map (colormap) of spectral contributions to highlight the modes of interest in a straightforward way (Figure 5). The color range corresponds to the square rooted intensity ratio of the selected simulated bands to the total intensity of modeled spectrum at any given wavenumber *ν*_i_ and additionally proportionalized to the calculated intensity at that point. The yielded value ranges from 0 (no contribution) to 1 (the NIR spectrum is influenced by the selected mode/modes entirely). The corresponding color varies from black to white, and it may be directly interpreted as the intensity of a given mode at a given wavenumber; the colortable is presented underneath the figure. The square root allows for elucidating less pronounced contributions. The density maps that are determined for various selections of modes-of-interest allow for unequivocal and thorough analysis of the influential determinants in the NIR spectrum of crystalline melamine (Figure 5), while keeping the figure compact and easy to read.

The upper NIR region (ca. 6900–6500 cm^−1^) is almost entirely populated by the first overtones and the binary combination bands of NH_2_ stretching modes (Figure 5). Other than this exception, the overtone bands of melamine may be described as non-essential for the other regions of NIR spectrum. Instead, the combination bands (binary combinations the most, ternary combinations to a bit lesser degree) play the primary role there. Again, the combinations that involve stretching NH_2_ modes are the most important factor. The corresponding binary combinations provide very strong influence in the 6900–6500 cm^−1^ region and throughout a broad 5100–4000 cm^−1^ region. Ternary combinations that involve NH_2_ stretching modes only give weak influence in the region of 6500–6000 cm^−1^, where the spectral lineshape is rather flat. The highly populated region between 5100–4000 cm^−1^ is mostly influenced by the combinations of stretching modes with deformation modes of NH_2_ groups. The other influential factor is *ν*C-N mode (both in ring as well as between C atoms in ring and NH_2_ groups). However, the wagging NH_2_ modes are largely suppressed. The modes that do not influence NIR region are as follows; overtones of *δ*_ip_ring, *δ*_ip_C-N(H_2_), *δ*_oop_ring, *δ*_oop_C-N(H_2_), *δ*_rock_NH_2_, *δ*_twist_NH_2_, *δ*_wagg_NH_2_.

The analysis of the NIR modes reveals a structural pattern. The modes that involve in-plane atoms displacements tend to impact NIR spectrum relatively stringer than those that involve out-of-plane motions. This observation will be discussed in detail in Section 2.4, together with the conclusions drawn from the analysis of IR spectrum of melamine (Section 2.1).

### 2.4. The Relationships Between IR and NIR Bands, and the Structural Features of Crystalline Melamine

Important conclusions may be drawn from the detailed comprehension of both IR and NIR spectra of crystalline melamine. The vibrational modes involving out-of-plane atomic motions are much more essential in the IR region (Figure 3 and Table 1), and thus a proper reflection of the inter-plane structure of crystalline melamine is required. Therefore, calculations that are based on a 3D infinite model of crystal lattice allows for the best description of these vibrations, even with harmonic approximation. However, in the NIR spectrum the most influential modes are those that involve in-plane atomic displacements. Thus, the neighboring planes of crystalline lattice do not impact these motions as much. Therefore, the NIR modes calculated for a single molecule are largely enough to accurately reflect the experimental spectrum.

This also makes perfect sense by comparing the IR vibrations calculated in periodic system to the ones calculated for a single molecule (also refer to Table S1 in Supporting Materials presenting the potential energy distribution calculated for melamine). In the calculations that are based on a single molecule (vs. the model of crystalline lattice), the out-of-plane deformation vibrations (e.g., *δ*_twist_NH_2_, *δ*_wagg_NH_2_, *δ*_oop_ring) are positioned at higher calculated wavenumbers, while the in-plane deformation vibrations are positioned at the lower calculated wavenumbers. Thus, the oscillators with the out-of-plane atomic motions have lower calculated force constants because of neglecting the surrounding crystalline planes. The neglecting of intra-plane interactions of melamine lead to a surprisingly miniscule decrease of the accuracy of the calculated NIR modes. The corresponding vibrations with in-plane atomic motions (primarily *ν*NH_2_, but also *δ*_ip_ring, *ν*C-N[H_2_]) were mostly overestimated in the calculated frequencies, which was easily corrected by applying scaling. These modes are seemingly less affected by the intermolecular interactions of melamine in crystal lattice, including the effects of hydrogen-bonding.

### 2.5. New Insights on the Quantitative Analytical Spectroscopy of Melamine

Melamine is known food adulterant, and for this reason, it has been frequently focused on analytical IR and NIR spectroscopy [9,42,43,44,45,46]. After reproducing the spectra of melamine, we can more deeply discuss some of the observations that were reported in these previous contributions.

NIR bands of melamine remain at relatively similar positions throughout various kinds of samples, unlike its IR bands, which are prone to shifting in response to the chemical environment [43]. This can be fully confirmed by our conclusions from quantum mechanical calculations. Lu et al. [42] have reported that the most relevant spectral region of milk powder useful for the detection of melamine is 5300–4900 cm^−1^. This region contains the binary and ternary combinations of NH_2_ stretching with NH_2_ deformation modes of melamine; however, no overtone bands can be found there (Figure 5). Cantor et al. [9] have found that the most meaningful region for the analysis of melamine content in gelatin by IR spectroscopy is at around 800 cm^−1^; the melamine bands therein stem from *δ*_oop_ring, *δ*_twist_NH_2_, and *δ*_wagg_NH_2_ (Figure 3 and Table 1). On the other hand, the chemometric models that are based on NIR spectra of gelatin contaminated by melamine have been found to recognize the most the 7000–6500 cm^−1^ region. The NIR bands of melamine due to the first overtones and binary combinations of both symmetric and asymmetric NH_2_ stretching modes can be exclusively found there (Figure 5). Our study allows for correcting Haughey et al. [45], who ascribed the NIR region of melamine in the vicinity of 6800 cm^−1^ to only overtone bands.

In more detail, the influential spectral regions in chemometric analysis of melamine content in milk powders based on NIR spectroscopy has been reported by Lim et al. [46]. They have found that the partial least squares regression (PLSR) models have recognized 6763 and 6808 cm^−1^ as the most meaningful in the analysis. They have concluded that these regions correspond to 6676 and 6529 cm^−1^ peaks of melamine observed in the spectrum of milk powder. We may now confirm that these two bands arise from the first overtones and binary combinations of *ν*_as_NH_2_ and *ν*_s_NH_2_ modes of melamine, respectively. These previous chemometric analyses consistently indicate that for the detection and quantification of melamine, its NH_2_ stretching vibrations are the most meaningful; however, we may now indicate that not only the first overtones, but also binary combinations equally contribute. On the other hand, the lower NIR region, where combination bands due to NH_2_ stretching and deformation modes of melamine are present, is less essential.

Balabin et al. [43] have concluded that, for the samples with high content of melamine, NIR spectroscopy yields better analytical performance than IR spectroscopy. It may be now confirmed that this occurs due to relatively much lower sensitivity of NIR bands to intermolecular interactions with the matrix molecules when compared to IR bands. The hydrogen-bonding influences the IR region of melamine much stronger than the NIR region, in which only minor band shifts and negligible intensity variations occur due to this effect. In such sense, this feature of pure melamine in crystal evidenced here (lower sensitivity of NIR bands than IR bands to the chemical environment of molecules of melamine), seems to also be universally preserved in complex samples consisting of various other molecules and biomolecules, e.g., in milk powder [43]. Thus, analytical IR and NIR spectroscopy can evidence the structural and vibrational properties of melamine that were unveiled in our study to have a direct impact on the detection and quantification of melamine content.

## 3. Materials and Methods

### 3.1. Experimental

Melamine was purchased from Alfa Aesar (A11295; ≥99% purity) and used without further purification. The measurement of IR spectra was carried out on a Perkin Elmer Spectrum 100 FT-IR spectrometer that was equipped with an ATR accessory and a diamond prism (PerkinElmer, Inc. Waltham, MA, USA). The spectrometer was controlled by Perkin Elmer Spectrum software (version 10.4.00). The spectra were measured in the 4000–650 cm^−1^ region with a spectral resolution of 4 cm^−1^. The number of accumulated scans was 16, and the measurement procedure was triplicated. The measurement was carried out at room temperature (~298 K). Sample preparation and spectra recording procedures were triplicated for each sample.

The measurement of NIR spectra was performed on a Büchi NIR Flex N-500 FT-NIR benchtop spectrometer that was controlled by the manufacturer’s NIRWare 1.4.3010 software (BUCHI^®^ AG, Flawil, Switzerland). The spectrometer was equipped with Büchi accessory for solid samples and operated in diffuse reflectance (DRIFT) mode and at room temperature (~298 K). The following recording parameters were selected; spectral resolution, spectral range, and scan number were 8 cm^−1^ (resulting in the interpolated 4 cm^−1^ of data spacing and 2 cm^−1^ of absolute accuracy), 10,000–4000 cm^−1^, and 64, respectively. Sample preparation and spectra recording procedures were triplicated for each sample.

The experimental spectra in this work presented adequate quality for qualitative assessment, with no need for preprocessing of any kind.

### 3.2. Quantum Mechanical Calculations

#### 3.2.1. IR spectrum Calculation in 3D Periodic Approximation

The simulation of IR spectra of crystalline melamine was based on harmonic analysis that was executed in three-dimensional periodic representation of crystal structure in Crystal 09 software (Aethia Srl, Italy) [59]. An infinite 3D model of crystal lattice of melamine was constructed by defining the primitive cell, in accordance with the experimental structural data obtained from the Cambridge Structural Database (CSD) [60,61]. An unconstrained and full geometry optimization was performed, in which both the atomic centers and cell parameters underwent the treatment prior to all subsequent calculations. The following procedural parameters were set throughout the geometry and vibrational computing steps. The Monkhorst–Pack reciprocal space was sampled over a shrinking factor that was equal to eight. The self-consistent field (SCF) direct procedure was iteratively converged with a tolerance of 10^−13^ atomic units per unit cell; the truncation of Coulomb and exchange sums in direct space was controlled by setting the Gaussian overlap tolerance criteria to 10^−8^, 10^−8^, 10^−8^, 10^−8^, and 10^−16^. With the objective of accelerating the convergence of the SCF procedure, a linear mixing of Fock matrices by 25% between adjacent steps and an energy shifting of 0.8 hartree for the first SCF cycle were employed. The electron integrals were numerically calculated over a dense (XL) integration grid. The periodic Density Functional Theory (DFT) computations were performed with the use of B3LYP (Becke, three-parameter, Lee-Yang-Parr) [62] single-hybrid density functional, as implemented in Crystal 09 software.

Two separate calculations each using different basis sets were carried out. In the first one, we applied the following basis sets for the respective atomic centers: 3-1p1G for hydrogen, and 6-31d1G for carbon and nitrogen. Since Gatti et al. have jointly proposed these basis sets [63], for sake of clarity in the present work we will refer to them as “Gatti” basis sets. For the second calculation, we used triple-ζ valence basis set with polarization (TZVP), applied uniformly for all atomic centers (C, N, H). The employment of the TZVP basis set substantially increased the computational expense. Section 2.1 discusses the quality of IR simulation obtained at B3LYP/TZVP and B3LYP/Gatti levels.

Harmonic vibrational frequencies and intensities were obtained at the Gamma point in each case. The frequencies are numerically obtained in Crystal 09; to ensure the high stability of this procedure, numerical derivation (in the calculation of the second derivatives of the potential energy) was based on two-point finite difference scheme. The convergence criterion for the vibrational analysis was successfully achieved, as the sonic modes of the crystal lattice approached near zero values (not exceeding −0.5 cm^−1^). The calculated band positions were overestimated; the overestimation was decreasing towards lower wavenumbers. Few factors contribute to this; the key ones are likely overestimation of bond strengths, imperfect molecular structure, and neglecting of anharmonicity. To account for this fact, the calculated wavenumbers were scaled while using linear scaling (Equation (1)).

(1) $$\nu_{scal}=\nu_{calc}-s\nu_{calc}^{2}$$

The best fit was achieved with the scaling parameter *s* equal to 2.7 × 10^−5^ (in the 4000–2500 cm^−1^ region) and 1.6 × 10^−5^ (in the 2500–650 cm^−1^ region). This scheme resembles the Wavenumber Linear Scaling (WLS), as developed by Yoshida et al. [64] Adjustments were necessary to fit the need of the study in a crystalline state. The calculated NIR spectra were scaled accordingly, while using scale factor *s* equal to 5.1 × 10^−6^ (Equation (1)). NIR scaling was significantly lesser; this is reasonable as the anharmonicity of the corresponding vibrations was already accounted for in the calculations. This is further explained in detail elsewhere (refer to Results and Discussion Section).

#### 3.2.2. Anharmonic Calculation of NIR Spectra

The prediction of NIR bands requires multi-modal anharmonic vibrational analysis. The methods that were based on Vibrational Second-order Perturbation Theory (VPT2) offer superior cost/accuracy factor therein [65,66,67]. In this work, we employed and compared the deperturbed (DVPT2) and generalized (GVPT2) variants [66]. The implementation of the latter one allowed for simulating second overtones and ternary combination bands. For the determination of the basic electronic properties at the DFT level, B3LYP functional and triple-*ζ* SNST basis set [67] were selected; this method has repeatedly been evidenced to deliver good results [31,33,65]. Long-range interactions were refined by Grimme’s D3 variant of empirical correction for dispersion with Becke–Johnson damping (GD3BJ) [68]. Prior to vibrational analysis, the molecular geometry optimization procedure was performed with very tight convergence criteria. The calculations were performed with the use of Gaussian 16 A.03 software [69].

The final spectra were simulated with the band broadening being obtained through a four-parameter Lorentz–Gauss product function [34,70] being applied to the quantum mechanically calculated IR and NIR band positions and intensities. The necessary data processing and assembly of figures were carried out in MATLAB R2016b [71].

## 4. Conclusions

Quantum mechanical calculations of IR spectra of crystalline melamine were successfully carried out for an infinite (3D periodic) lattice model. All of the experimental IR bands were accurately reproduced, even when using reasonably affordable harmonic approximation. On the other hand, finite models gave very inaccurate calculated IR spectra. In contrast, the NIR spectrum of crystalline melamine was accurately modeled by anharmonic calculations that are based on the model of a single molecule. It is a striking difference in the dependence of the quality vs. model complexity, between these two spectral regions. This result may be explained based on direct comparison of the molecular motions corresponding to the vibrations being influential for the IR and NIR regions.

Vibrations involving out-of-plane atomic motions strongly affect the IR region of melamine. Therefore, it is essential to incorporate a proper description of the inter-plane interactions of melamine molecules as they appear in the crystalline lattice. The neglecting of the neighboring molecules in proximity planes leads to a completely incorrect calculated IR spectrum. In contrast, the in-plane vibrations of melamine are less affected by inter-molecular interactions. The vibrations involving in-plane atomic displacements are the most essential for the NIR spectrum of melamine. On the other hand, the out-of-plane motions are either suppressed in intensity or they are located outside of the NIR region. Hence, the calculated NIR spectrum of melamine is not significantly affected by a radical simplification of the molecular model. Even a single molecule model provides accurate reproduction of NIR spectrum of crystalline melamine. It may be concluded that the long-range ordering and, in particular, inter-plane interactions in the crystal lattice of melamine are significantly less important factors for NIR modes than for IR modes. From this observation, another important conclusion may be drawn. Due to the very significant computational cost of anharmonic calculations, the possibility to reduce the complexity of the molecular model (i.e., non-necessity to use 3D infinite model, which is also computationally costly) in the simulation of NIR spectra offers promising possibilities for similar studies of other crystalline materials.

Our findings shed light on the spectral features of melamine that have been reported in analytical spectroscopic studies of melamine as contaminant. In particular, the concluded in literature superiority of NIR spectroscopy in the analysis of the samples with relatively higher content of melamine was explained.

## Figures and Tables

**Table 1:** Band assignments in the experimental ATR-IR spectrum of crystalline melamine. Band numbers correspond to those presented in Figure 3A,B.

Band Number	Wavenumber/cm^−1^			Assignment
	Experimental	Scaled Calc.	Non-Scaled Calc.	
1	3468	3454	3669	*ν*_as_NH_2_
2	3416	3423	3634	*ν*_as_NH_2_
3	3324	3296	3491	*ν*_as_NH_2_
4	~3188	3252	3441	*ν*_as_NH_2_
5	3121.7	3179	3360	*ν*_s_NH_2_
6	1647.7	1627	1705	*δ*_sciss_NH_2_
7	1624.9	1599	1675	*δ*_sciss_NH_2_
8	~1574	1553	1624	*δ*_sciss_NH_2_; *δ*_ip_ring
9	1527.7	1528 1523	1597 1591	*δ*_sciss_NH_2_; *δ*_rock_NH_2_; *δ*_ip_ring *δ*_rock_NH_2_; *δ*_ip_ring
10	1465.6	1449	1511	*δ*_sciss_NH_2_; *ν*C-N(H_2_)
11	1431.6	1417	1476	*ν*C-N(H_2_); *δ*_ip_ring
12	1194.3	1197	1238	*δ*_rock_NH_2_
13	1173.5	1177 1167	1217 1206	*δ*_rock_NH_2_
14	1024.1	1035 1021	1066 1051	*δ*_rock_NH_2_; *δ*_ip_ring *δ*_rock_NH_2_; *ν*C-N(H_2_); *δ*_ip_ring
15	810.1	811 801	830 819	*δ*_oop_ring; *δ*_twist_NH_2_ *δ*_wagg_NH_2_
16	768.4	755	771	*δ*_twist_NH_2_
17	674.5	661	673	*δ*_wagg_NH_2_

## Supplementary Materials

The following are available online at https://www.mdpi.com/1420-3049/24/7/1402/s1, Figure S1: Optimized (B3LYP\Gatti) primitive cell of melamine. CRYSTAL output (A–D), and with atoms wrapped to the cell (E–H). Figure S2: Comparison of the experimental ATR-IR spectrum of polycrystalline melamine (4000–2500 cm^−1^ region) with the spectra calculated for periodic (3D, infinite) model of crystal lattice in harmonic approximation at B3LYP/Gatti and B3LYP/TZVP levels. Figure S3: Comparison of the experimental ATR-IR spectrum of polycrystalline melamine (2000–650 cm^−1^ region) with the spectra calculated for periodic (3D, infinite) model of crystal lattice in harmonic approximation at B3LYP/Gatti and B3LYP/TZVP levels. Table S1: Projection of normal coordinates of melamine onto natural internal coordinates. Based on Potential Energy Distribution (PED) analysis carried out for single molecule model vibrationally analyzed at B3LYP-GD3BJ/SNST level.
