# Supplementary material for: Distinct Difference in Sensitivity of NIR vs. IR Bands of Melamine to Inter-Molecular Interactions with Impact on Analytical Spectroscopy Explained by Anharmonic Quantum Mechanical Study
Source: Molecules. 2019 Apr 10;24(7):1402. doi: 10.3390/molecules24071402 (PMC6479474; doi:10.3390/molecules24071402)
Supplement: Supplementary file 1 [file molecules-24-01402-s001.pdf]

molecules

MDPI

Supplementary Materials

Distinct Difference in Sensitivity of NIR vs. IR Bands of Melamine to Inter-molecular Interactions with Impact on Analytical Spectroscopy Explained by Anharmonic Quantum Mechanical Study

Supplementary Materials

Justyna Grabska [1], Krzysztof B. Beć [1,2*], Christian G. Kirchler [1], Yukihiro Ozaki [3], and Christian W. Huck [1]

[1] Institute of Analytical Chemistry and Radiochemistry, Leopold-Franzens University, Innrain 80/82, CCB-Center for Chemistry and Biomedicine, 6020-Innsbruck, Austria; justyna.grabska7@gmail.com (J.G.); Christian.Kirchler@uibk.ac.at (C.G.K.); Christian.W.Huck@uibk.ac.at (C.W.H.)

[2] Faculty of Chemistry, University of Wrocław, F. Joliot-Curie 14, 50-383 Wrocław, Poland

[3] Department of Chemistry, School of Science and Technology, Kwansei Gakuin University, Sanda, Hyogo 669-1337, Japan; ozaki@kwansei.ac.jp (Y.O.)

* Correspondence: krzysztof.bec@chem.uni.wroc.pl.

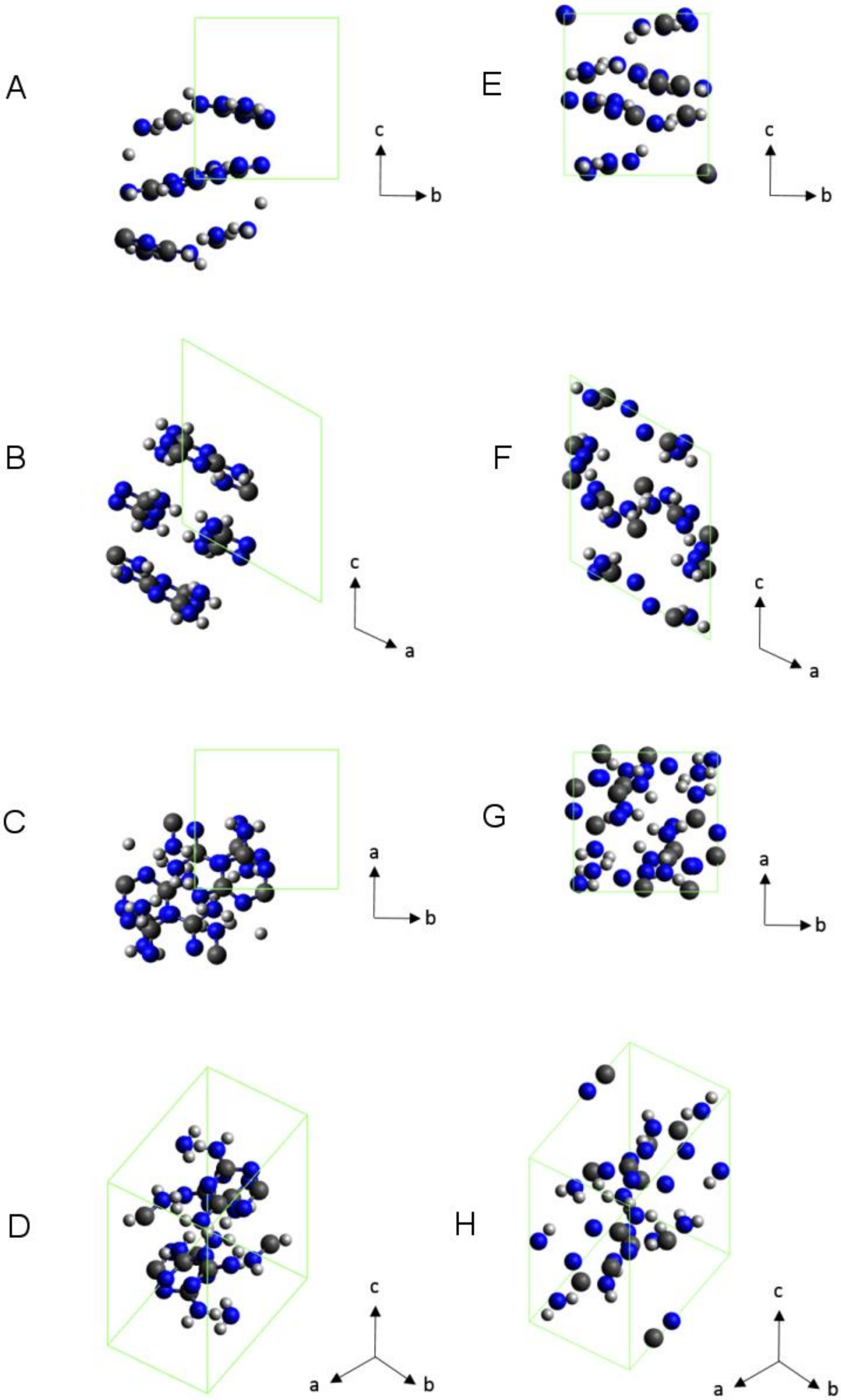

Figure S1. Optimized (B3LYP\Gatti) primitive cell of melamine. CRYSTAL output (A-D), and with atoms wrapped to the cell (E-H).

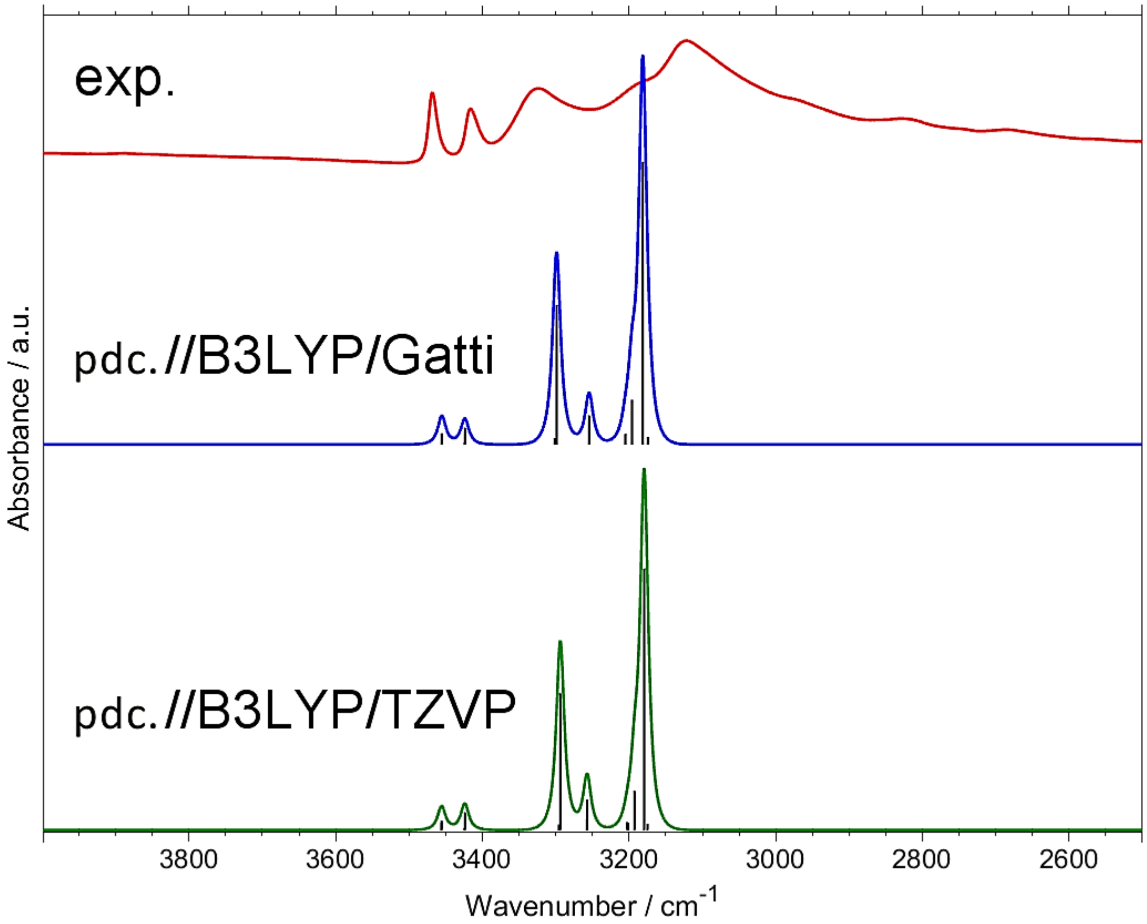

Figure S2. Comparison of the experimental ATR-IR spectrum of polycrystalline melamine (4000-2500 cm^{-1} region) with the spectra calculated for periodic (3D, infinite) model of crystal lattice in harmonic approximation at B3LYP/Gatti and B3LYP/TZVP levels.

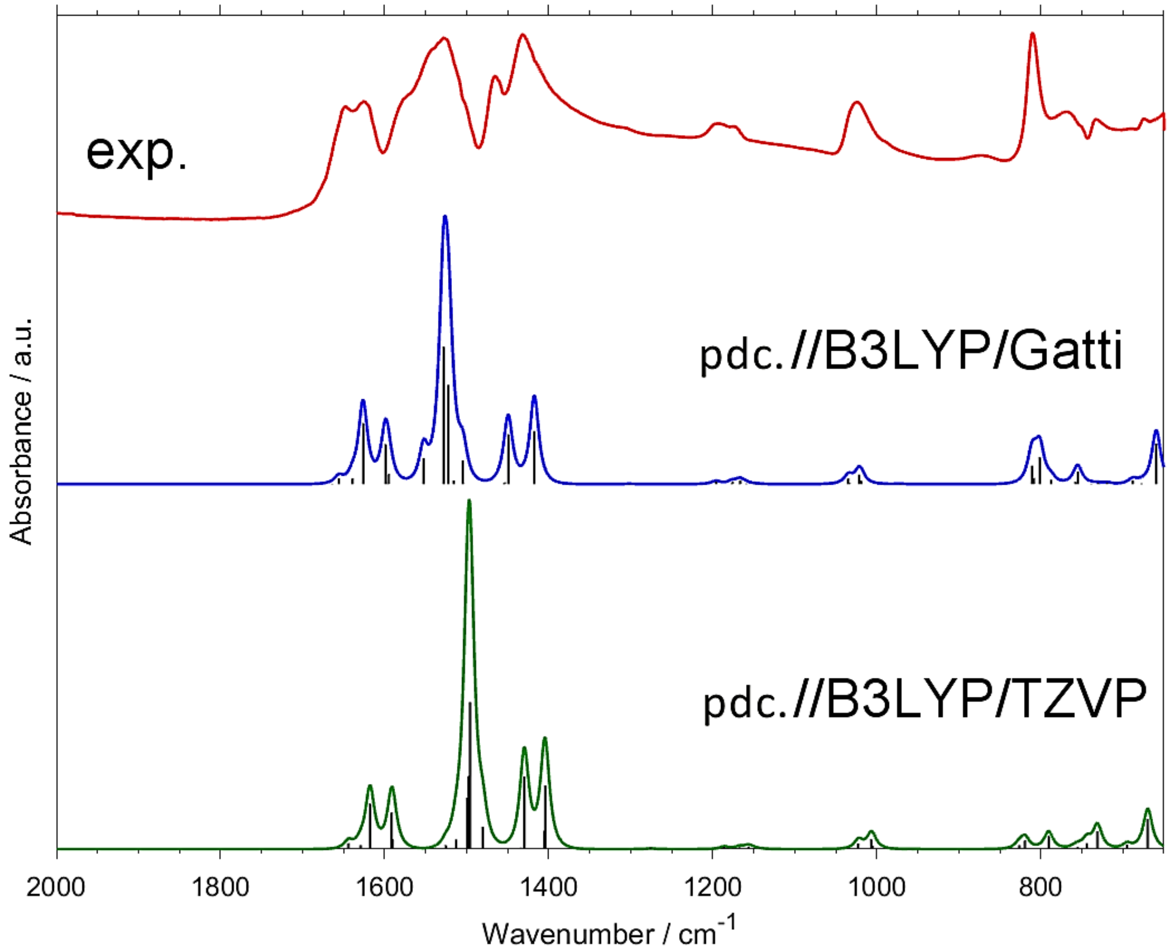

Figure S3. Comparison of the experimental ATR-IR spectrum of polycrystalline melamine (2000-650 cm^{-1} region) with the spectra calculated for periodic (3D, infinite) model of crystal lattice in harmonic approximation at B3LYP/Gatti and B3LYP/TZVP levels.

Table S1. Projection of normal coordinates of melamine onto natural internal coordinates. Based on Potential Energy Distribution (PED) analysis carried out for single molecule model vibrationally analyzed at B3LYP-GD3BJ/SNST level.

Calc. Freq. [cm^{-1}]		Contributions of Natural Internal Coordinates							
Harm	Anharm								
3728.7	3591.9	**100%**	ν_{as} NH_2 in-phase (C_1, C_3)						
3728.4	3591.5	**100%**	ν_{as} NH_2 out-of-phase (C_1, C_3)						
3725.5	3585.3	**100%**	ν_{as} NH_2 (C_5)						
3595.4	3467.9	**92%**	ν_s NH_2						
3594.0	3467.1	**100%**	ν_s NH_2 out of phase (C_1, C_3)						
3592.3	3463.0	**92%**	ν_s NH_2 (C_5)						
1658.2	1616.0	**45%**	δ_{sciss} NH_2 in phase	**39%**	νC-N(H_2)	**11%**	δ_{ip} ring		
1617.5	1578.3	**50%**	δ_{sciss} NH_2	**18%**	νC-N(H_2)	**12%**	νC-N (ring)	**5%**	δ_{ip} ring
1617.4	1577.0	**50%**	δ_{sciss} NH_2	**12%**	νC-N(H_2)	**14%**	νC-N (ring)	**6%**	δ_{ip} ring
1596.0	1539.5	**58%**	νC-N (ring)	**22%**	δ_{sciss} NH_2	**5%**	δ_{ip} ring		
1595.8	1541.4	**60%**	νC-N (ring)	**14%**	δ_{sciss} NH_2	**6%**	δ_{ip} ring		
1511.0	1473.8	**48%**	δ_{sciss} NH_2	**24%**	νC-N(H_2)	**19%**	δ_{ip} ring		
1465.2	1432.3	**34%**	νC-N(H_2)	**26%**	νC-N (ring)	**14%**	δ_{sciss} NH_2	**5%**	δ_{ip} ring
1464.8	1430.6	**40%**	νC-N (ring)	**35%**	νC-N(H_2)	**9%**	δ_{sciss} NH_2	**6%**	δ_{ip} ring
1304.6	1256.0	**84%**	νC-N (ring)						
1172.1	1139.8	**38%**	νC-N(H_2)	**20%**	δ_{rock} NH_2	**16%**	νC-N (ring)		
1171.3	1138.2	**38%**	νC-N(H_2)	**28%**	δ_{rock} NH_2	**26%**	νC-N (ring)		

1147.9	1095.9	**59%**	δ_{rock} NH_2	**37%**	νC-N (ring)		
1002.1	958.3	**48%**	δ_{rock} NH_2	**34%**	νC-N (ring)	**6%**	δ_{ip} ring
1000.9	954.7	**50%**	δ_{rock} NH_2	**34%**	νC-N (ring)	**6%**	δ_{ip} ring
989.5	971.3	**47%**	δ_{ip} ring	**48%**	νC-N (ring)		
831.2	817.8	**52%**	δ_{oop} ring	**47%**	δ_{oop} C-N(H_2)		
748.4	742.8	**61%**	δ_{oop} C-N(H_2)	**34%**	δ_{oop} ring		
747.7	741.8	**65%**	δ_{oop} C-N(H_2)	**34%**	δ_{oop} ring		
686.7	683.3	**45%**	νC-N(H_2)	**26%**	δ_{ip} ring		
588.6	583.0	**78%**	δ_{ip} ring	**6%**	δ_{ip} C-N(H_2)	**5%**	νC-N(H_2)
588.4	578.9	**77%**	δ_{ip} ring				
558.3	516.3	**48%**	δ_{twist} NH_2	**27%**	δ_{ip} C-N(H_2)		
533.7	541.7	**92%**	δ_{twist} NH_2				
530.1	507.5	**97%**	δ_{twist} NH_2				
508.7	576.6	**64%**	δ_{twist} NH_2	**18%**	δ_{rock} NH_2		
337.9	328.4	**82%**	δ_{ip} C-N(H_2)				
337.8	331.8	**83%**	δ_{ip} C-N(H_2)	**5%**	δ_{rock} NH_2		
293.1	289.9	**77%**	δ_{wagg} NH_2	**13%**	νC-N(H_2)		
275.3	274.2	**76%**	δ_{wagg} NH_2	**12%**	νC-N(H_2)		
252.3	248.5	**76%**	δ_{wagg} NH_2	**14%**	νC-N(H_2)		
205.5	204.2	**66%**	δ_{oop} C-N(H_2)	**34%**	δ_{oop} ring		
184.3	175.9	**51%**	δ_{oop} ring	**46%**	δ_{oop} C-N(H_2)		
183.6	164.7	**53%**	δ_{oop} C-N(H_2)	**43%**	δ_{oop} ring		

Funding:

This work was supported by the Austrian Science Fund (FWF), P32004-N28.

This work was supported by the National Science Center Poland (NCN), Grant 2017/27/B/ST4/00948.

Acknowledgements:

Calculations have been carried out in Wrocław Centre for Networking and Supercomputing (http://www.wcss.pl), under grant no. 375.

Conflicts of Interest: The authors declare no conflict of interest. The funders had no role in the design of the study; in the collection, analyses, or interpretation of data; in the writing of the manuscript, or in the decision to publish the results.
